# Cluster Validity Index for Uncertain Data Based on a Probabilistic Distance Measure in Feature Space

**DOI:** 10.3390/s23073708

**Published:** 2023-04-03

**Authors:** Changwan Ko, Jaeseung Baek, Behnam Tavakkol, Young-Seon Jeong

**Affiliations:** 1 Department of Industrial Engineering, Chonnam National University, Gwangju 61186, Republic of Korea; kcw7536@gmail.com; 2 College of Business, Northern Michigan University, Marquette, MI 49855, USA; jbaek@nmu.edu; 3 Department of Industrial & Systems Engineering, Rutgers University, Piscataway, NJ 08854, USA; 4 School of Business, Stockton University, Galloway, NJ 08205, USA; behnam.tavakkol@stockton.edu; 5 Interdisciplinary Program of Arts and Design Technology, Chonnam National University, Gwangju 61186, Republic of Korea

**Keywords:** uncertain data, cluster validity index, kernel probabilistic distance, feature space

## Abstract

Cluster validity indices (CVIs) for evaluating the result of the optimal number of clusters are critical measures in clustering problems. Most CVIs are designed for typical data-type objects called certain data objects. Certain data objects only have a singular value and include no uncertainty, so they are assumed to be information-abundant in the real world. In this study, new CVIs for uncertain data, based on kernel probabilistic distance measures to calculate the distance between two distributions in feature space, are proposed for uncertain clusters with arbitrary shapes, sub-clusters, and noise in objects. By transforming original uncertain data into kernel spaces, the proposed CVI accurately measures the compactness and separability of a cluster for arbitrary cluster shapes and is robust to noise and outliers in a cluster. The proposed CVI was evaluated for diverse types of simulated and real-life uncertain objects, confirming that the proposed validity indexes in feature space outperform the pre-existing ones in the original space.

## 1. Introduction

The purpose of clustering is to partition objects into groups with criteria such that the similarity within the groups and the dissimilarity among different groups should be maximized [1,2]. Although clustering methods have been widely used in many applications, most clustering algorithms do not provide the optimal number of clusters. Partitional-based clustering algorithms such as K-means clustering [3] must preset the number of clusters [4]. As cluster information is rarely known in the real world, it is crucial to evaluate the clustering results depending on the different numbers of clusters. Although many clustering methods exist for diverse applications, such as pattern recognition [5], semiconductor manufacturing [6], and healthcare [7], they have been developed primarily for only certain data or fixed values. However, the embedded uncertainty of data is essential in many applications. For instance, a patient’s blood pressure may not be consistent because of environmental conditions and instrument errors. Furthermore, measurement values are continuously changing because of the positions of instrumentation devices or workers’ conditions. Aside from these examples, data randomness, missing data, delayed updates, and worker fatigue are other factors of data uncertainty [8,9].

**Figure 1:**
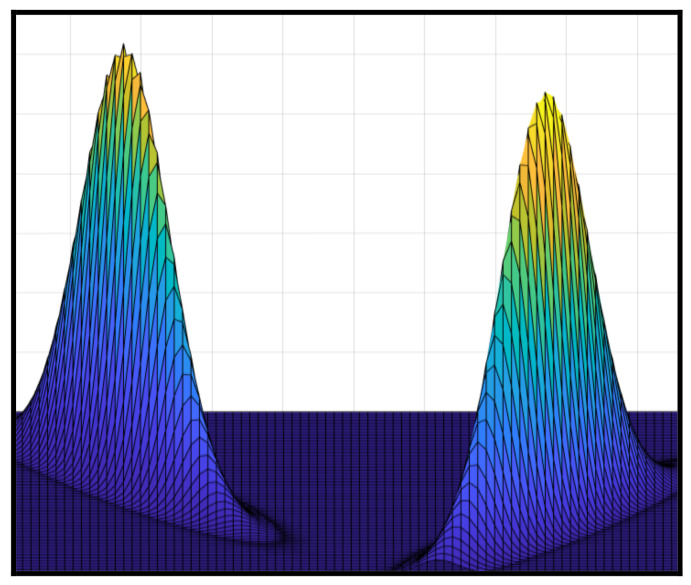
Two uncertain datasets, each expressed by a PDF.

Uncertain data are assumed to be prevalent information in the real world, e.g., measurement errors and environmental conditions. The uncertainty of uncertain data can be expressed by probability density functions (PDFs). Figure 1 illustrates two uncertain data, each distributed by a PDF. The standard method of converting uncertain data is to transform a summary statistic (e.g., mean or median) into certain data. However, these statistics could lose extra information of uncertainty that is significant to capture the uncertainty information of uncertain objects. 

Cluster validity indices (CVIs), which are indicators for validating the quality of clustering algorithms, have been widely used to determine the correct number of clusters for the given data. As the CVIs only use input data information, they must be used according to the characteristics of the data. The two components of a CVI are compactness and separability measures. The former refers to an intra-cluster distance, and the latter represents an inter-cluster distance. Most CVIs indicate that a good partition produces a small compactness value and a high separability value. However, the existing CVIs are vulnerable to validating cluster results when the shapes of the clusters are not spherical clusters [10,11].

For certain data, several CVIs, such as the Dunn [12], Calinski–Harabasz [13], Davies–Bouldin [14], and Xie–Beni [15] indices, have been proposed based on combinations of compactness and separability measures. However, most of the existing CVIs have been developed for certain data. There have been few studies on uncertain data. Moreover, relatively new CVIs are also being designed to incorporate mathematical theories into pre-existing CVIs, such as the K-nearest neighbor algorithm, which is used to compute compactness and separation by taking into account shared/non-shared data pairs [10], and principal component analysis, which is used to capture the geometry of the clusters [16]; or to develop clustering algorithms to cluster more well-separated clusters [1]. 

To apply uncertain data to the existing CVIs’ formulas, they should be changed to calculate distance measures of compactness and separability. In a study of uncertain CVIs, Tavakkol et al. [17] proposed CVIs for uncertain data to calculate the distance between two uncertain objects using probabilistic distance measures in the original space. However, it leads to sensitivity to arbitrary shapes of clusters, sub-clusters, and outliers because of the clusters shape that may cause inaccurate compactness and separability [11].

**Figure 2:**
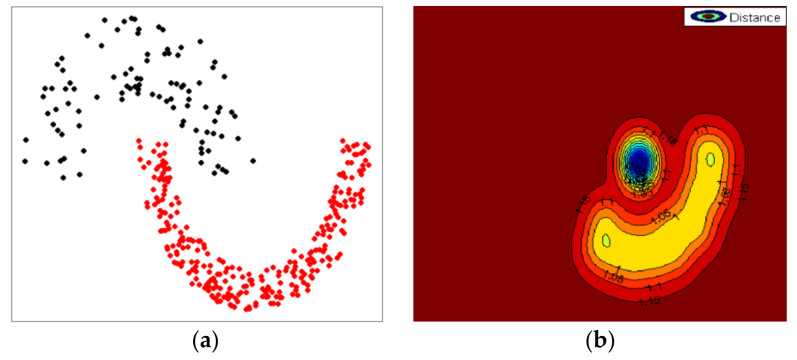
Visualization of kernel transformation: (**a**) asymmetry shape in original space; (**b**) transformed shape in feature space.

Consequently, this study proposes new uncertain CVIs for uncertain data objects based on kernel probabilistic distance measures in feature space. The proposed CVIs for uncertain objects are designed to adapt the kernel-based Bhattacharyya probabilistic distance in kernel spaces. In kernel space, the proposed CVIs produce accurate compactness and separability for the arbitrary shapes of clusters by transforming them into elliptical shapes in feature space. Figure 2 illustrates that the ambiguous shape of a dataset in the original space is transformed into a relatively elliptical, circular shape in feature space; thus, the kernel transformation can improve performance in calculating accurate compactness and separability. Furthermore, the proposed approaches could be robust to noise and outliers in a cluster. The superior performance of the proposed CVIs was evaluated through diverse experiments, including simulated and real-life datasets.

This paper is organized as follows. Section 2 reviews the previous studies on CVIs. New CVIs for uncertain data based on a kernel probabilistic distance measure are proposed in Section 3. After the extensive experiments are presented in Section 4, the conclusions and future studies are provided in Section 5.

## 2. Related Work

### 2.1. CVI for Certain Data

In the past few decades, many CVIs have been developed to determine the optimal number of clusters. Most CVIs focus on calculating compactness and separability measures. The combination of the two measures is composed of a ratio-type or summation-type index. This section presents several popular CVIs that have been evaluated in many applications. 

The Dunn (DU) index [12]:

(1) $$DU_{K}=\frac{min_{i,j=1,\cdots ,K,\;i\neq j}\left\{min_{x\in C_{i},\;\;y\in C_{j}}d\left(x,y\right)\right\}}{max_{i=1,\cdots ,K}\left\{max_{x,y\in C_{i}}d\left(x,y\right)\right\}}.$$

Compactness and separability are computed using the maximum diameter among all clusters and the minimum pair-wise distance between objects in different clusters. The DU index is integrated by the ratio type of separability to compactness. Thus, the maximum value of the DU index is the optimal number of clusters (max. S/C). 

Calinski–Harabasz (CH) index [13]:

(2) $$CH_{K}=\frac{\sum_{i=1}^{K}n_{i}\cdot d{\left(z_{i}\cdot z_{tot}\right)}^{2}}{K-1}\cdot \frac{n-K}{\sum_{1=1}^{K}\sum_{x\in C_{i}}d{\left(x,z_{i}\right)}^{2}}$$

The CH is composed of the ratio type of separability and compactness like the DU index. $z_{tot}$ is the centroid of the entire dataset. Compactness and separability are computed using within- and between-cluster sums of squares. Thus, the maximum value for CH is the optimum partition (max. S/C).

The Davies–Bouldin (DB) index [14]:

(3) $$DB_{K}=\frac{1}{K}\underset{i=1,\;\ldots ,K,\;i\neq j}{\max}\left\{\left(\sqrt{\frac{1}{n_{i}}\sum_{x\in C_{i}}d{\left(x,z_{i}\right)}^{2}}+\sqrt{\frac{1}{n_{j}}\sum_{y\in C_{j}}d{\left(y,z_{j}\right)}^{2}}\right)/d\left(z_{i},z_{j}\right)\right\}$$

where $z_{i}$ and $z_{j}$ are the centroids of each cluster. Compactness and separability are calculated using the sum of mean squares of individual clusters, unlike the DU index, which considers the compactness and separability of the total cluster. Compactness is the computed sum of the pair-wise distances between different clusters; separability is calculated differently for each cluster. The DB index is comprised of the ratio types of compactness and separability. Therefore, the minimum value of DB is the optimum partition (min. C/S).

The pre-existing CVIs are sensitive to sub-clusters, arbitrary shapes, and noise in clusters for the compactness measure [18]. This study overcomes those drawbacks by conducting a spatial transformation from the original space into feature space using a kernel function that correctly measures cluster compactness and separability.

### 2.2. CVI for Uncertain Data

Most CVIs have focused on certain data or fixed values [19]. Certain data do not have uncertainty caused by several factors and environments such as sensor measurement error, repeated measurements by workers, or equipment operating environments. Uncertain data objects come in two possible forms: (1) multiple points for each object and (2) a PDF for each object, either given or obtained by fitting the multiple points [20]. Several studies related to clustering uncertain data have been conducted. However, CVIs for uncertain data have rarely been used. The CVIs are crucial criteria for validating the results of clusters [21,22] to find the appropriate number of clusters. Therefore, the study of CVIs for uncertain data is necessary.

In this study, the proposed CVIs use kernel probabilistic distance measures to compute the distance between two uncertain data objects. There are many popular probabilistic distance measures, such as Bhattacharyya distance [23], Wasserstein distance, and Kullback–Leibler divergence [24]. This study uses the Bhattacharyya distance measure. The Bhattacharyya distance measure is one of the widely used probabilistic distance measures and has been generally used in diverse applications. 

The Bhattacharyya distance between two probability distributions can be calculated in discrete and continuous cases. Let p and q be the continuous probability distributions over the same space. The definition of the Bhattacharyya distance for a continuous case in original space can be described as follows:

(4) $$PD_{Bhatt}\left(p,q\right)=-\ln\left\{\;\int_{x}\sqrt{p\left(x\right)q\left(x\right)}dx\right\}$$

There are closed-form solutions for many probabilistic distance measures, including the Bhattacharyya distance, for cases where uncertain data objects are modeled with multivariate normal distributions. As probabilistic distance measures can capture the distance between PDFs, they can also be used to capture the distance between uncertain data objects [25]. The Bhattacharyya distance is a special case of Chernoff distance with parameters $\alpha_{1}=\alpha_{2}=1/2$, and the closed-from of Bhattacharyya distance for multivariate normal PDFs is defined in Equation (5):

(5) $$PD_{Bhatt}\left(p,q\right)=\frac{1}{8}{\left(\mu_{p}-\mu_{q}\right)}^{\prime }{\left(\Sigma_{p}+\Sigma_{q}\right)}^{-1}\left(\mu_{p}-\mu_{q}\right)+\frac{1}{2}\ln\left(\frac{\left|\Sigma_{p}+\Sigma_{q}\right|}{2{\left(\left|\Sigma_{p}\left|+\right|\Sigma_{q}\right|\right)}^{\frac{1}{2}}}\right)$$

where $\mu_{p}$ and $\mu_{q}$ are means, and $\Sigma_{p}$ and $\Sigma_{q}$ are covariance matrices of $P\;\sim \;MVN\left(\mu_{p},\;\Sigma_{p}\right)$ and Q$\;\sim \;MVN\left(\mu_{q},\;\Sigma_{q}\right)$. 

This study models the Bhattacharyya distance between two uncertain data objects in kernel space. We can compute the probabilistic distance between two uncertain data objects in feature space using a kernel function.

## 3. Proposed CVIs for Uncertain Data

### 3.1. Kernel Probabilistic Distance Measure in Feature Space

Computing the probabilistic distance is a nontrivial problem. We can compute the Bhattacharyya distance in feature space by referring to several steps developed by Zhou and Chellappa [26]. In capturing the probabilistic distance, suppose that $x_{1}=\left\{x_{11},x_{21},\ldots ,x_{N1}\right\}$ and $x_{2}=\left\{x_{12},x_{22},\ldots ,x_{N2}\right\}$ are the given objects in original space ${\mathbb{R}}^{d}$ with a multivariate normal density function:

(6) $$N\left(x;\;\mu ,\;\Sigma \right)=\frac{1}{\sqrt{{\left(2\pi \right)}^{d}\left|\Sigma \right|}}\exp\left\{-\frac{1}{2}{\left(x-\mu \right)}^{T}\Sigma^{-1}\left(x-\mu \right)\right\}$$

The radial basis function (RBF) kernel function displayed in Equation (7) can be used to transfer original data into feature space for calculating the distance between uncertain data objects $x_{1}$ and $x_{2}$. The RBF kernel function is commonly used in various fields and algorithms because it outperforms other kernel functions [27,28].

(7) $$K_{ij}=\exp\left(-\frac{1}{2\sigma^{2}}\|x_{i}-x_{j}\|{}^{2}\right),\;i,j=1,2$$

In kernel function $K\left(x_{1},\;x_{2}\right)$, where $x_{1},\;x_{2}\in {\mathbb{R}}^{d}$, and the non-linear mapping function ϕ and kernel Gram matrix K are defined as $K=\Phi^{T}\Phi$, where $\Phi :={\;\Phi }_{N}=\left[\phi \left(x_{1}\right),\;\phi \left(x_{2}\right),\;\ldots ,\;\phi \left(x_{N}\right)\right]\in {\mathbb{R}}^{f}$, and f≫d represents the data transformed to kernel space. The mean μ and covariance matrix Σ in feature space are estimated as:

(8) $$\mu =N^{-1}\overset{N}{\underset{n=1}{\sum }}\phi \left(x_{n}\right)=\Phi ,$$

(9) $$\Sigma =N^{-1}\sum_{n=1}^{N}\left(\phi_{n}-\mu \right){\left(\phi_{n}-\mu \right)}^{T}=\Phi JJ^{T}\Phi^{T},$$

where $J=\frac{1}{\sqrt{n}}$($I_{N}-s\vec{1}$) with $s_{N\times 1}=\frac{1}{N}{\vec{1}}^{T}$ and $\vec{1}=\left[1,1,\;\ldots ,\;1\right]$.

The covariance matrix Σ must be converted into approximation form because of its rank-deficient characteristic f≫d. Therefore, we can use the approximation form as follows:

(10) $$C=\Phi JJ^{T}\Phi^{T}+\rho I_{f}=WW^{T}+\rho I_{f}=\Phi A\Phi^{T}+\rho I_{f},$$

where $W\dot{=}\;\Phi JQ$, $A\dot{=}JQQ^{T}J^{T}$, and ρ is a user parameter that should be pre-specified in advance. 

Obtaining the matrix Q requires computing the top r eigenvalues matrix $\Lambda_{r}$ and the top r eigenvectors matrix $V_{r}$ of $\bar{K}=J^{T}KJ$, where top r is a pre-specified parameter; thus, r=3 is used. Q is an N×r matrix calculated as follows:

(11) $$Q\dot{=}V_{r}{\left(I_{r}-\rho \Lambda_{r}^{-1}\right)}^{1/\;2}.\;$$

Define matrix P as:

(12) $$P_{\left(N_{1}+N_{2}\right)\times \left(r_{1}+r_{2}\right)}=\left[\begin{array}{cc} \sqrt{\alpha_{1}}J_{1}Q_{1} & 0\\ 0 & \sqrt{\alpha_{2}}J_{2}Q_{2} \end{array}\right].\;$$

The Bhattacharyya distance is a special case of Chernoff distance; it must be set to $\alpha_{1}=\alpha_{2}=1/2$ for all experiments. The $\tau_{i},i=1,\ldots ,r_{1}+r_{2}$, are eigenvalues of a $L_{ch}$ matrix, with dimensions of $\left(r_{1}+r_{2}\right)\times \left(r_{1}+r_{2}\right)$ given by

(13) $$L_{ch}=P^{T}\left[\begin{array}{c} \Phi_{1}^{T}\\ \Phi_{2}^{T} \end{array}\right]\left[\Phi_{1}^{T}\;\Phi_{2}^{T}\right]P=P^{T}\left[\begin{array}{cc} K_{11} & K_{12}\\ K_{21} & K_{22} \end{array}\right]P.\;$$

Scalar values $\varepsilon_{11},\;\varepsilon_{12},\;\varepsilon_{22}$ are computed by Equation (14).

(14) $$\varepsilon_{ij}=s_{i}^{T}K_{ij}s_{j}-s_{i}^{T}\left[K_{i1}K_{i2}\right]B_{ch}\left[\begin{array}{c} K_{1j}\\ K_{2j} \end{array}\right]s_{j}$$

where $B_{ch}=P{\left(\rho I_{r_{1}+r_{2}}+L_{ch}\right)}^{-1}P^{T}$ with dimensions of $\left(N_{1}+N_{2}\right)\times \left(N_{1}+N_{2}\right)$. 

The kernel-based probabilistic Bhattacharyya distance between two uncertain data objects $x_{1}$ and $x_{2}$ in feature space is calculated as follows:

(15) $$KPD_{Bhatt}=0.5[\alpha_{1}\alpha_{2}\rho^{-1}\left(\varepsilon_{11}+\varepsilon_{22}-2\varepsilon_{12}\;\right)+0.5\sum_{i=1}^{r_{1}+r_{2}}\log\frac{\rho +\tau_{i}}{\lambda_{i,1}}+\sum_{i=1}^{r_{1}+r_{2}}\log\frac{\rho +\tau_{i}}{\lambda_{i,2}}\;,\;$$

where $\lambda_{i,j\;}$, $i=1,\ldots ,r_{j}$ are the eigenvalues of $C_{j}$:

(16) $$\lambda_{i,j}=\left\{\begin{array}{ll} \lambda_{i,j}, & when\;i=1,\ldots ,r_{j}\\ \rho , & when\;i=r_{j}+1,\ldots ,r_{1}+r_{2} \end{array}\right.\;$$

### 3.2. New CVI for Uncertain Data 

The uncertain data objects in the cluster are transformed into feature space to compute the compactness and separability in the feature space by applying a kernel function. The mapped uncertain data objects are used to compute the distance between different clusters for calculating compactness and separability, which are used to obtain the values of the proposed CVIs. The calculated value of the indices changes according to the number of clusters K, and the proposed uncertain feature space DU (UFSDU) and uncertain feature space CH (UFSCH) index, are defined in Equations (17) and (18), respectively:

UFSDU index:

(17) $$UFSDU_{K}=\frac{min_{i,j=1,\cdots ,K,\;i\neq j}\left\{min_{x\in C_{i},\;\;y\in C_{j}}KPD_{Bhatt}\left(x,y\right)\right\}}{max_{i=1,\cdots ,K}\left\{max_{x,y\in C_{i}}\;KPD_{Bhatt}\left(x,y\right)\right\}}$$

UFSCH index:

(18) $$UFSCH_{K}=\frac{\sum_{i=1}^{K}n_{i}\cdot KPD_{Bhatt}{\left(z_{i}\cdot z_{tot}\right)}^{2}}{K-1}\cdot \frac{n-K}{\sum_{i=1}^{k}\sum_{x\in C_{i}}KPD_{Bhatt}{\left(x,z_{i}\right)}^{2}}$$

These proposed CVI equations are similar to the DU and CH indices, except for the term $KPD_{Bhatt}\left(x,y\right)$, which is the computed distance between two uncertain data objects in feature space in Equation (15).

## 4. Experimental Results

In this study, we propose two CVIs that are calculated probabilistic distances between different uncertain data objects in feature space. The K-medoids clustering algorithm proposed by Jiang et al. [19] was used to compare the performances of the proposed CVIs in feature space. The K-medoids algorithm is one of the most useful algorithms in clustering problems, which uses probabilistic distance measures to capture the similarity between uncertain objects. It differs from the popular K-means clustering algorithm used for clustering data into groups in its robustness to outliers. The K-means method represents each cluster by the mean of all objects in this cluster, whereas the K-medoids method calculates the distance between every pair of all uncertain data objects and the medoid within a cluster [19]. Then, of all calculated distance values, uncertain data with the smallest distances are assigned as a new medoid for the cluster. We proceeded with the experiments by setting the value of K, which is the number of clusters and is used as the probabilistic distance measure. In this study, we varied the number of clusters (K) and the Bhattacharyya distance measure to compute distances between different uncertain data objects in feature space. 

### 4.1. Experimental Procedure for Uncertain Data

Experiments were performed with artificial and real-world datasets that may have sub-clusters and clusters with asymmetrical, arbitrary, and noisy shapes to evaluate the performances of the proposed CVIs. A normalization process was conducted for each feature of the datasets to reduce the scale gap between different features defined in Equation (19):

(19) $$x_{norm}=\frac{x-x_{min}}{x_{max}-x_{min}},\;$$

where $x_{min}$ and $x_{max}$ are the minimum and maximum values of one feature of the dataset. We then simulated uncertain data objects from certain data objects by following the methodology used by [20].

The pre-existent DU and CH indexes were used to compute uncertain data objects in original space—uncertain original space, DU (UOSUD), and uncertain original space, CH (UOSCH)—to confirm the validity of the proposed CVIs. The overall experimental procedure is represented by Algorithm 1. The procedure used to compare the performances of the proposed CVIs with those of the previous CVIs was as follows: The inputs included the number of uncertain data objects N, the number of object features M, and the number of clusters K. We modeled the uncertain data with multivariate normal distributions. The means of the distributions were the original certain data. The covariances were estimated as follows:

(20) $$f\left(S_{i}^{k}|\Psi^{k},df^{k}\right)=\frac{{\left|\Psi^{k}\right|}^{\frac{df^{k}}{2}}}{\frac{p\cdot df^{k}}{2}\Gamma_{p}\left(\frac{df^{k}}{2}\right)}{\left|S_{i}^{k}\right|}^{-\frac{df^{k}+p+1}{2}}e^{-\frac{1}{2}tr\left(\Psi^{k}{\left(S_{i}^{k}\right)}^{-1}\right)},\;\;i=1,\ldots ,n_{k},\;k=1,\ldots ,K$$

where $S_{i}^{k}$ represents the covariance matrices for objects in class k with the inverse Wishart PDF [29], as defined in Equation (20) [20]. $\Psi^{k}$ is a positive definite scale matrix and $df^{k}$ is the degree of freedom. p indicates the dimensions of $S_{i}^{k}$, $tr\left(\cdot \right)$ is the trace of a matrix, and Γ is the multivariate gamma function.
**Algorithm 1:** K-medoids for uncertain data using a probabilistic distance measure in feature space.
1.  **Input**: n: The number of objects in cluster k, K: The number of clusters, iter = 0;
2.  Randomly select the cluster medoids $C^{\left(0\right)}=\{c_{1}^{\left(0\right)\;},\ldots ,c_{K}^{\left(0\right)}\}$ obtained from the initial clusters
3.  Initialize 
4.  $CVIs=\left\{cvi^{\left(1\right)},\;\ldots ,\;cvi^{\left(K\right)}\right\}$ obtained UOSDU, UOSCH, UFSDU, and UFSCH
5.  **Repeat**

6.  **for**
k=2 to K

7.    $c_{k}^{\left(old\right)}=c_{k}^{\left(0\right)}$; $c_{k}^{\left(new\right)}=0$

8.    Compute the new medoids: 
9.    **while** $c_{k}^{\left(old\right)}\;\neq c_{k}^{\left(new\right)}$ 
10.      $p=\underset{1\leq i\leq n}{\underbrace{argmin}}\overset{k}{\underset{j=1}{\sum }}KPD_{Bhatt}(x_{i},\;c_{jk}^{\;})$, where j is an index of cluster medoid in $c_{k}^{\;}$ 
11.      $c_{k}^{\left(new\right)}=x_{p}$ 
12.    **end**

13.    Calculate the $cvi^{\left(k\right)}$ using Equations (1), (2), (17), and (18). 
14.  **end**

15.  iter = iter + 1 
16. **Until** (iter = Maxiter)

Step 1: Set K initial clusters with uncertain objects randomly for a given dataset. Run a K-medoids clustering algorithm with different values for the K parameter (2 ≤ K ≤10).

Step 2: Obtain the medoids of each cluster for which the sum of the probabilistic distance between the objects is the smallest.

Step 3: Calculate CVIs for all the partitions. We calculated the compactness and separability in kernel space using an RBF kernel function with σ (bandwidth in the RBF kernel function). The optimal value was determined through a set of preliminary experiments by taking [0.1, 0.2, …, 4] in σ. 

Step 4: We increased the reliability of experimental results by replicating the experiment 100 times for the same dataset with different trial seeds to obtain the initial medoids in Step 1 and used the average value of CVI for each cluster. 

Step 5: Finally, we evaluated each CVI and the suggested number of clusters from a CVI; the actual numbers of clusters of a dataset were then compared.

### 4.2. Experiments with Artificial and Real-World Datasets 

Experiments were conducted to evaluate the proposed CVIs in comparison to the pre-existent CVIs. These experiments used 10 datasets with sensitive characteristics containing arbitrariness, sub-clusters, asymmetry, and noise provided by the UCI (https://archive.ics.uci.edu/, accessed on 10 March 2023) [30] and Tomas Barton repositories (https://github.com/deric/clustering-benchmark, accessed on 10 March 2023), which have 122 artificial datasets with arbitrariness, sub-clusters, and asymmetric shapes in two or three features. The datasets from UCI repository, (e.g., D3, D4, D5, and D7) were collected in real environmental conditions; however, the other datasets were artificially created, which can be checked in Tomas Barton repositories.

**Figure 3:**
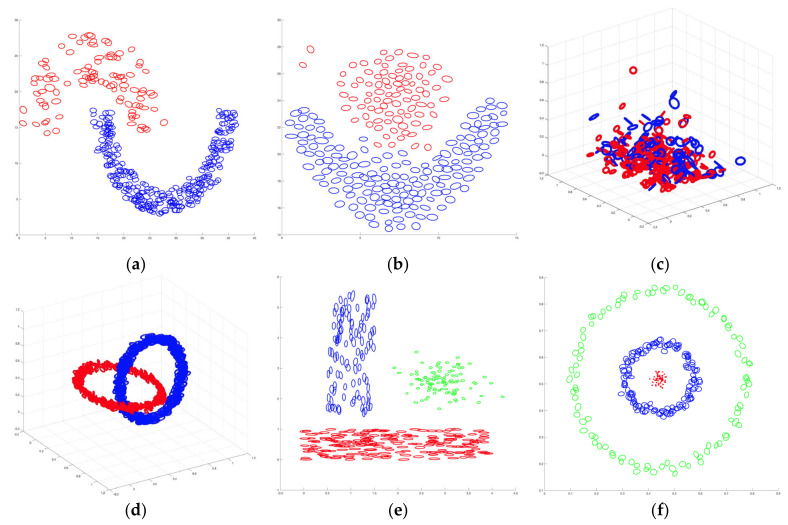
Shapes of 2D and 3D datasets: (**a**) D1 dataset; (**b**) D2 dataset; (**c**) D7 dataset; (**d**) D8 dataset; (**e**) D9 dataset; (**f**) D10 dataset.

The summary of datasets used for the experiments is presented in Table 1. Two-dimensional (2D) and three-dimensional (3D) dataset shapes are illustrated in Figure 3. The CVI values were computed by changing the number of clusters (K) in each dataset and then comparing the predicted labels of experiments to the actual labels in the datasets. 

### 4.3. Performance Comparison of the Proposed CVIs 

The experimental results are given in Table 2, Table 3, Table 4, Table 5, Table 6, Table 7, Table 8, Table 9, Table 10 and Table 11. The actual number of clusters is below the name of the dataset. It is also noted with an asterisk (*) adjacent to the actual number of clusters along the top. Moreover, all the results of the datasets are presented in Table 12, indicating the performance of the proposed CVIs by a quantitative figure. Each cell in Table 12 represents the optimal number of clusters K determined by its CVI criteria.

The bold values with gray-shaded backgrounds indicate the optimal cluster K decided by each CVI. As presented in Table 2, three of the CVIs succeeded in estimating the number of clusters as two in D1. UOSCH failed. The proposed UFSDU and UFSCH also successfully predicted the number of clusters in D2. In contrast, UOSDU failed to estimate the number of clusters in D2. 

Although the proposed UFSDU index and the pre-existent CVIs failed to predict the number of clusters in D3, UFSCH was successful. All CVIs correctly predicted the number of clusters for some datasets; see Table 5, Table 7 and Table 8. In contrast, the proposed UFSDU index is the only CVI that correctly predicted the actual number of clusters in D5, as presented in Table 6. Furthermore, the UFSDU index predicted the actual number of clusters of D8. D8’s shape (Figure 3) is classified distinctly into two classes when viewed visually. However, it is challenging to calculate the compactness and separability of a cluster in the original space. Nevertheless, the UFSDU index was successful in such predictions; the UFSCH forecasted the number of clusters as three, which is close to the actual number of clusters, two. The kernel transformation facilitates computation to obtain greater compactness and separability in the feature space than the original space, leading to high-performance clustering.

The UOSCH index and the new CVIs predicted the number of clusters to be three in D9, and the UOSDU and UFSCH indexes successfully estimated the number of clusters in D10. Table 12 presents a summary of the results of the 10 datasets above, whereas the symbol of a circled dot (⨀) indicates that the CVI accurately predicted the actual number of clusters. As presented in Table 12, the pre-existent CVIs precisely estimated the number of clusters for five experimental datasets, whereas the newly proposed CVIs accurately predicted the number of clusters for eight datasets—three more than the pre-existent CVIs. 

## 5. Conclusions

In this study, we proposed novel cluster validity indices (CVIs) for uncertain data objects in feature space. Unlike conventional CVIs in original space, the proposed CVIs are used for uncertain data objects with arbitrariness, sub-clusters, and noisy shapes of clusters that are hard to evaluate, by transforming the uncertain data from the original space to the feature space, which is performed by the kernel function. The proposed CVIs measure the compactness and separability of each cluster in kernel space, which transforms the original data into a higher-dimensional space, leading to less sensitivity to the arbitrary shapes of clusters and more robustness to noise and outliers. We compared the performances of the proposed CVIs with those of pre-existent CVIs that only consider for the original space. The Bhattacharyya distance measure, one of the most widely used for calculating distance, was used to perform experiments with several artificial and real-life datasets to capture the distances between probability density functions. Numerical examples, including a real-life case study and artificial datasets, confirmed that our proposed CVIs are robust to arbitrary cluster shapes, especially sub-clusters, and are promising alternatives for evaluating the fitness of clustering results that can find the optimal number of clusters, K. The proposed CVIs outperform the pre-existent CVIs because of the application of kernel functions to uncertain data, transforming them from the original space to the feature space. As for practical significance, the proposed CVIs could be utilized in diverse applications. For example, Kim et al. proposed new a multivariate kernel density estimator for uncertain data classification for mixed defect patterns on DRAM wafer maps [31]. The proposed CVI method could be applied for evaluating the number of defect patterns on wafer maps. However, there are some limitations to the proposed CVIs. The uncertain data are assumed to have multivariate normal distributions in advance to compute the distances between different uncertain data objects. The uncertainty of the uncertain data may have a variety of probability functions (normal distribution, exponential distribution, etc.), and some cannot be strictly modeled by PDFs. This might be overcome through methods for generating random variables and support-measure data description, which is a non-parametric machine learning method that does not require an assumption of a prior distribution to be made in advance.

Future research should consider the compactness measure in kernel space in advanced machine learning algorithms, such as support vector data descriptions or Bayesian frameworks of Bayesian support vector data descriptions. The concepts of our CVIs can also be applied to other clustering algorithms.

## Figures and Tables

**Table 1:** Summary of datasets.

Dataset Index	Dataset Name	# of Obs.	# of Dim.	# of Clusters	Projection Shape
D1	A.K Jain’s Toy	373	2	2	Asymmetry, Arbitrary shape
D2	Flame	240	2	2	Sub-cluster, Noise
D3	Iris	150	4	3	-
D4	Thyroid	215	5	2	-
D5	Wine	178	13	3	-
D6	Wisconsin	683	9	2	-
D7	Harberman	301	3	2	Random shape
D8	Chainlink	1000	3	2	Sub-cluster, Arbitrary shape
D9	Lsun	400	2	3	Asymmetry, Arbitrary shape
D10	Zelnik1	299	2	3	Sub-cluster

**Table 2:** Performance results for D1.

		# of Clusters	2 *	3	4	5	6	7	8	9	10
	CVI										
D1 (2)	UOSDU		**0.00075**	0.00063	0.00049	0.00046	0.00043	0.00047	0.00044	0.00042	0.000410
	UOSCH		554.4796	537.8279	573.5387	**586.5310**	576.5872	562.0666	575.2021	566.6556	567.6008
	UFSDU		**0.011830**	0.00727	0.007410	0.006350	0.006920	0.006390	0.006740	0.00580	0.005630
	UFSCH		**256.0945**	204.767	167.9338	149.5915	138.3076	128.206	122.4676	117.0263	112.4593

**Table 3:** Performance results for D2.

		# of Clusters	2 *	3	4	5	6	7	8	9	10
	CVI										
D2 (2)	UOSDU		0.00578	0.00581	0.00583	**0.00533**	0.00494	0.00494	0.00452	0.00454	0.00448
	UOSCH		**218.9052**	188.6698	201.7685	195.0877	190.2412	190.7961	192.3785	187.7774	186.0032
	UFSDU		**0.01875**	0.01433	0.01619	0.01386	0.01284	0.01261	0.01263	0.0125	0.01271
	UFSCH		**246.7711**	190.3472	184.7522	163.52	150.3938	143.1108	138.9139	131.6189	127.3284

**Table 4:** Performance results for D3.

		# of Clusters	2	3 *	4	5	6	7	8	9	10
	CVI										
D3 (3)	UOSDU		**0.57393**	0.18691	0.06671	0.04599	0.03375	0.03045	0.02475	0.02443	0.02427
	UOSCH		**393.8149**	340.7616	288.9103	257.4766	227.8328	211.7321	193.9894	179.4227	172.1492
	UFSDU		**0.78121**	0.05291	0.0332	0.02818	0.0201	0.02217	0.02033	0.01676	0.01503
	UFSCH		97.24412	**100.9677**	83.47847	74.68629	65.08186	59.80128	55.42499	51.32508	48.54411

**Table 5:** Performance results for D4.

		# of Clusters	2 *	3	4	5	6	7	8	9	10
	CVI										
D4 (2)	UOSDU		**0.01059**	0.00702	0.00447	0.00389	0.00338	0.00285	0.0029	0.00264	0.00254
	UOSCH		**52.44662**	49.27229	45.29772	44.23136	46.29286	43.05835	40.0334	38.99379	36.43862
	UFSDU		**0.09045**	0.02678	0.02097	0.01941	0.0186	0.0166	0.01728	0.01604	0.01577
	UFSCH		**88.16833**	63.62494	54.54528	48.32164	43.33752	38.65073	35.53446	32.89777	30.6346

**Table 6:** Performance results for D5.

		# of Clusters	2	3 *	4	5	6	7	8	9	10
	CVI										
D5 (3)	UOSDU		**0.28546**	0.19218	0.16953	0.13451	0.13042	0.12188	0.1222	0.11775	0.11498
	UOSCH		**46.98845**	41.61822	34.08324	29.45127	26.66111	23.71564	21.97848	20.8878	19.0692
	UFSDU		0.1351	**0.13992**	0.12361	0.11102	0.1058	0.10544	0.10343	0.10402	0.10242
	UFSCH		**166.5115**	94.11775	70.17926	57.15066	48.48219	42.44803	38.19718	34.55733	31.1674

**Table 7:** Performance results for D6.

		# of Clusters	2 *	3	4	5	6	7	8	9	10
	CVI										
D6 (2)	UOSDU		**0.10223**	0.04719	0.02262	0.01209	0.00742	0.00342	0.0014	0.00109	0.00075
	UOSCH		**237.829**	186.8503	145.4631	119.3866	98.36381	89.72379	80.18472	70.83073	66.12163
	UFSDU		**0.22631**	0.10763	0.04928	0.03902	0.01416	0.01228	0.0084	0.00605	0.00391
	UFSCH		**349.3685**	261.4169	205.8692	171.2457	144.4285	124.5582	109.2653	97.50292	88.98401

**Table 8:** Performance results for D7.

		# of Clusters	2 *	3	4	5	6	7	8	9	10
	CVI										
D7 (2)	UOSDU		**0.00198**	0.0014	0.00112	0.00086	0.00078	0.00089	0.00069	0.00079	0.00076
	UOSCH		**128.8359**	117.8517	104.8203	97.56451	95.82686	92.17925	86.85381	84.98897	82.71107
	UFSDU		**0.13021**	0.02577	0.01681	0.01199	0.01108	0.01122	0.01132	0.01028	0.00945
	UFSCH		**319.3255**	171.7169	127.0638	104.5919	90.63319	80.94442	72.86994	67.51974	62.62473

**Table 9:** Performance results for D8.

		# of Clusters	2 *	3	4	5	6	7	8	9	10
	CVI										
D8 (2)	UOSDU		0.00019	0.00017	0.00017	0.00017	0.00017	0.00018	**0.00021**	0.00019	0.00017
	UOSCH		419.8882	371.9768	388.8548	430.2229	426.5956	430.8854	**449.3122**	438.7834	417.3569
	UFSDU		**0.00439**	0.00237	0.00204	0.00114	0.0013	0.00118	0.00149	0.00153	0.0014
	UFSCH		445.5408	**463.2664**	449.4758	439.8262	425.4487	411.5018	422.1565	428.8755	437.9047

**Table 10:** Performance results for D9.

		# of Clusters	2	3 *	4	5	6	7	8	9	10
	CVI										
D9 (3)	UOSDU		**0.01277**	0.00168	0.00087	0.00081	0.00069	0.00062	0.0006	0.00063	0.00054
	UOSCH		316.7407	**406.3877**	395.188	401.578	380.968	363.1193	365.4242	349.9761	351.8199
	UFSDU		0.01439	**0.02006**	0.01697	0.01119	0.00658	0.00574	0.00485	0.00472	0.00416
	UFSCH		190.3465	**205.1745**	189.6315	175.6124	164.8462	154.2108	149.907	141.5702	133.6363

**Table 11:** Performance results for D10.

		# of Clusters	2	3 *	4	5	6	7	8	9	10
	CVI										
D10 (3)	UOSDU		0.030644	**0.049296**	0.048849	0.048798	0.046752	0.044594	0.042478	0.037749	0.041905
	UOSCH		**235.4205**	161.3342	142.117	135.4194	127.012	126.4954	125.6673	123.9964	132.4379
	UFSDU		**0.00368**	0.00123	0.00123	0.00115	0.00103	0.00087	0.00073	0.00077	0.00056
	UFSCH		102.6013	**106.5976**	99.7133	98.79822	97.68495	95.67929	95.82844	96.62246	102.6371

**Table 12:** Difference between the actual and estimated numbers of clusters in lower-dimensional datasets.

Dataset	Dim	# of Clusters	UOSDU	UOSCH	UFSDU	UFSCH
D1	2	2	⨀	5	⨀	⨀
D2	2	2	4	⨀	⨀	⨀
D3	4	3	2	2	2	⨀
D4	5	2	⨀	⨀	⨀	⨀
D5	13	3	2	2	⨀	2
D6	9	2	⨀	⨀	⨀	⨀
D7	3	2	⨀	⨀	⨀	⨀
D8	3	2	8	8	⨀	3
D9	2	3	2	⨀	⨀	⨀
D10	2	3	⨀	2	2	⨀
# of successes in estimating the optimal number of clusters			5	5	8	8

## Data Availability

The real-world datasets used in this study are available at: https://archive.ics.uci.edu/ml/index.php accessed on 10 March 2023; the artificial datasets that contain data sensitive to shapes are available at: https://github.com/deric/clustering-benchmark/tree/master/ accessed on 10 March 2023.

## Abbreviations

The following abbreviations are used in this manuscript:

Bhatt	Bhattacharyya distance measure
C/S	Separability/Compactness
CH	Calinski–Harabasz
CVIs	Cluster validity indices
DB	Davies–Bouldin
DU	Dunn
KPD	Kernel-based probabilistic distance
PD	Probabilistic distance
PDF	Probability density function
RBF	Radial basis function
S/C	Compactness/Separability
UFSCH	Uncertain feature space CH
UFSDU	Uncertain feature space DU
UOSCH	Uncertain feature space CH
UOSDU	Uncertain feature space DU
